# PPARgamma activation attenuates T-lymphocyte-dependent inflammation of adipose tissue and development of insulin resistance in obese mice

**DOI:** 10.1186/1475-2840-9-64

**Published:** 2010-10-18

**Authors:** Anna Foryst-Ludwig, Martin Hartge, Markus Clemenz, Christiane Sprang, Katharina Heß, Nikolaus Marx, Thomas Unger, Ulrich Kintscher

**Affiliations:** 1 Center for Cardiovascular Research (CCR), Institute of Pharmacology Charité - Universitätsmedizin Berlin, Germany; 2 Department of Internal Medicine I - University Hospital Aachen, Germany

## Abstract

**Background:**

Inflammation of adipose tissue (AT) has been recently accepted as a first step towards obesity-mediated insulin resistance. We could previously show that mice fed with high fat diet (HFD) develop systemic insulin resistance (IR) and glucose intolerance (GI) associated with CD4-positive T-lymphocyte infiltration into visceral AT. These T-lymphocytes, when enriched in AT, participate in the development of fat tissue inflammation and subsequent recruitment of proinflammatory macrophages. The aim of this work was to elucidate the action of the insulin sensitizing PPARgamma on T-lymphocyte infiltration during development of IR, and comparison of the PPARgamma-mediated anti-inflammatory effects of rosiglitazone and telmisartan in diet-induced obesity model (DIO-model) in mice.

**Methods:**

In order to investigate the molecular mechanisms underlying early development of systemic insulin resistance and glucose intolerance male C57BL/6J mice were fed with high fat diet (HFD) for 10-weeks in parallel to the pharmacological intervention with rosiglitazone, telmisartan, or vehicle.

**Results:**

Both rosiglitazone and telmisartan were able to reduce T-lymphocyte infiltration into AT analyzed by quantitative analysis of the T-cell marker CD3gamma and the chemokine SDF1alpha. Subsequently, both PPARgamma agonists were able to attenuate macrophage infiltration into AT, measured by the reduction of MCP1 and F4/80 expression. In parallel to the reduction of AT-inflammation, ligand-activated PPARgamma improved diet-induced IR and GI.

**Conclusion:**

Together the present study demonstrates a close connection between PPARgamma-mediated anti-inflammation in AT and systemic improvement of glucose metabolism identifying T-lymphocytes as one cellular mediator of PPARgamma´s action.

## Background

The prevalence of metabolic diseases such as obesity, type 2 diabetes and obesity-associated hypertension is increasing gradually[1]. Given that IR was shown to correlate with reduction of insulin-mediated glucose uptake in skeletal muscle and adipose tissue, IR was recently recognized as a key etiological factor of those metabolic disorders[2]. The molecular mechanisms underlying the development of obesity-directed IR are not well understood. Several lines of evidence support the thesis, that the first step towards the development of autonomous insulin resistance in adipose tissue, as well as in the liver, is inflammation[3-5].

Pro-inflammatory cytokines produced by adipocytes in fat tissue such as TNFalpha and IL-6 accelerate inflammatory responses of the surrounding tissue, and recruit pro-inflammatory cells. Recently we were able to show that the first inflammatory cells recruited to the adipose tissue during development of obesity-induced IR are CD4-positive T-lymphocytes[6]. The recruitment of T-lymphocytes into fat tissue is likely mediated through resident adipocytes expressing stromal cell-derived factor-1 alpha (SDF-1alpha), a known attractant molecule for T-cells. In parallel, adipocytes produce monocyte chemoatractant protein-1 (MCP-1) for subsequent attraction of macrophages. Then, CD4-positive T-lymphocytes are able to induce pro-inflammatory responses in macrophages by the release of interferon gamma (IFNgamma)[6,7]. It is well known that macrophages which are recruited during the development of the obesity-related IR to fat tissue belong to the "pro-inflammatory" M1-phenotype[3,8]. Several independent research groups have demonstrated, that M1 macrophages are highly activated, sensitive to lipopolysaccharide (LPS) and free fatty acids (FFA), express F4/80, CD11b and CD11c markers as well as toll like receptors (TLR) 2 and 4, and produce a wide range of pro-inflammatory cytokines, such as TNFalpha and IL-6. This is in contrast to resident anti-inflammatory M2 macrophages, with a low sensitivity to LPS and FFA, lack of CD11c marker, and production of anti-inflammatory cytokines such as IL-4 and IL-10[3,8,9]. Recently Stienstra and colleagues[10] reported the M2- to M1-transition of resident adipose tissue specific macrophages (ATM) in rodents fed with HFD. Additionally, the authors showed that the transition could be inverted in HFD-fed mice treated with the PPARgamma agonist rosiglitazone.

PPARgamma belongs to the nuclear hormone receptor family of transcription factors, which are activated upon binding of specific ligands or agonists. PPARgamma is a key regulator of glucose and lipid metabolism by controlling energy homeostasis in adipose tissue, liver and skeletal muscle[11]. Glitazones or Thiazolidinediones (TZDs), such as pioglitazone and rosiglitazone, are potent synthetic PPARgamma agonists used in clinic to treat type 2 diabetes[12]. The activation of PPARgamma leads to improvement of systemic IR/glucose tolerance and the metabolic function of adipose tissue, liver and skeletal muscle. Recently we could demonstrate that a subgroup of angiotensin type 1 receptor (AT1R) blockers (ARBs) such as telmisartan act as a partial PPARgamma agonists, and show - similar to full agonists-beneficial metabolic effects in mouse model of HFD-induced IR[13].

The aim of the present study was to elucidate the role of ligand-activated PPARgamma activation on T-lymphocyte-derived adipose tissue inflammation. Our data indicate that PPARgamma plays a central role in the development of IR, acting as an anti-inflammatory factor on T-lymphocyte activation and infiltration into fat tissue, and by this mean contributing to the attenuation of the systemic development of IR.

## Methods

### 1. Mice Model

Male C57BL/6J mice, 4-5 weeks of age, were purchased from Harlan Winkelmann (Borchen, Germany). All mice were housed in a temperature controlled (25°C) facility with a 12 h light/dark cycle. Mice were randomised to either a vehicle-treated (0.5% Tween80/H_2_O), rosiglitazone-treated (10 mg/kg/d), or telmisartan-treated (3 mg/kg/day) group (all applied by oral gavage) and fed with a high-fat diet (HFD (60% kcal from fat). Age-match low fat diet (LFD (10% kcal from fat)-fed mice served as controls [13] for 5- and 10-weeks respectively. To exclude differences induced by food intake, all HFD-fed mice were pair-fed. After 5- and 10-weeks on HFD animals were metabolically phenotyped including an intra-peritoneal glucose tolerance test (ipGTT) using a dose of 1 g/kg body weight glucose, and an insulin tolerance test (ITT) by injecting 0.25 U/kg body weight insulin (ActrapidHM, Novo Nordisk) intraperitoneally. Tail vein blood was used for glucose quantification during ipGTT and ITT using a Glucometer (Precision Xtra, Abbott). Afterwards, animals were sacrificed and organs were dissected. All animal procedures were in accordance with institutional guidelines and were approved.

### 2. Immunohistological staining

For immunohistological studies, gonadal adipose tissue was fixed in 4% formalin, embedded in paraffin and stained with the corresponding antibodies. The following antibodies were used: lymphocytes were identified by staining with an anti-mouse CD3gamma antibody (Serotec, MCA 1477) and macrophages were detected using Macrophage Marker (RM0029-11H3), a monoclonal antibody raised against isolated macrophages of mouse origin, (Santa Cruz, SC-101447). Control staining was performed using type and isomatched IgG antibodies.

### 3. Quantitative real-time PCR

Quantitative real-time PCR was performed as previously described[14]. Briefly, total RNA was isolated from gonadal adipose tissue using Trizol (Invitrogen) according to the manufacturer's instructions. The quantitative PCR reactions were carried out in the presence of a fluorescent dye (Sybrgreen, BioRad). Relative abundance of mRNA was calculated after normalisation to 18s ribosomal RNA. The expression of MCP1, F4/80, SDF-1alpha and CD3gamma were measured using assay on demand (Mm00441242-m1; Mm00802529-m1; Mm00445552-m1 and MM00438095 respectively, from Life Technologies).

## Results

### 1. Early stages of systemic insulin resistance and glucose intolerance are attenuated by ligand-mediated PPARgamma activation

In order to investigate the molecular mechanisms underlying early development of systemic insulin resistance and glucose intolerance male C57BL/6J mice were fed with high fat diet (HFD) for 10-weeks in parallel to the pharmacological intervention with rosiglitazone (10 mg/kg/d), telmisartan (3 mg/kg/d), or vehicle. Mice fed with low fat diet (LFD) were used as control (data not shown). Total body weight of the HFD-fed mice after 10-weeks of intervention was significantly increased in vehicle treated animals, when compared to LFD-fed littermates (data not shown). When treated with telmisartan, a partial PPARgamma agonist, HFD-fed mice showed a significant reduction of body weight (BW), apparent as soon as after 5-weeks of treatment (Table 1). Significant reduction of BW was also observed in rosiglitazone-treated HFD mice after 10-weeks of treatment, when compared to the vehicle treated group, which could be explain, at least in part, by the pair feeding protocol (Table 1). Food intake among vehicle-, telmisartan- and rosiglitazone-treated HFD-fed mice was similar (Table 2).

**Table 1:** Body weight development after 5 and 10 weeks of HFD

BW (g)	5 Weeks of HFD			10 Weeks of HFD	
Vehicle	24.8 ± 2.3			30.5 ± 2.5	
Rosi	24.7 ± 1.4	p < 0.05 vs. Telmi		27.8 ± 1.0	p < 0.05 vs. Veh p < 0.05 vs. Telmi
Telmi	23.6 ± 1.5	p < 0.05 vs. Veh		24.7 ± 1.2	p < 0.05 vs. Veh

**Table 2:** Food intake after 5 and 10 weeks of HFD (pair-feeding)

Food Intake (g/day/mouse)	5 Weeks of HFD			10 Weeks of HFD	
Vehicle	1.2 ± 0.1			1.1 ± 0.2	
Rosi	1.1 ± 0.6	ns		1.0 ± 0.1	ns
Telmi	1.1 ± 0.2	ns		1.0 ± 0.1	ns

**Figure 1:**
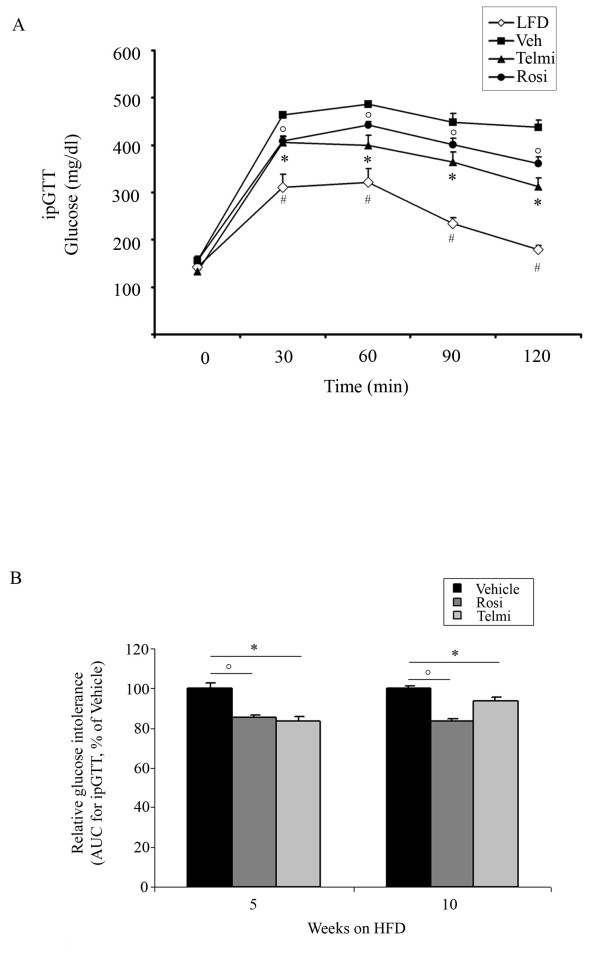
**PPARgamma activation improves systemic insulin sensitivity of HFD-fed mice**. A) Glucose tolerance test (GTT) of LFD-fed, HFD-fed and vehicle-, rosiglitazone-, or telmisartan-treated mice after 10-weeks of intervention, as indicated and described in Methods, n = 10; * p < 0.05 rosiglitazone-treated mice vs. vehicle-treated animals,°p < 0.05 telmisartan-treated mice vs. vehicle-treated animals, # p < 0.05 LFD-fed vs. HFD-fed, vehicle-treated mice. B) Glucose tolerance test (GTT) of HFD-fed, vehicle-, rosiglitazone-, or telmisartan-treated mice after 5- and 10-weeks of intervention, as indicated. From each group area under the curve (AUC) was calculated, as described in Methods, n = 10;°p < 0.05 rosiglitazone-treated mice vs. vehicle-treated animals; * p < 0.05 telmisartan-treated mice vs. vehicle-treated animals.

**Figure 2:**
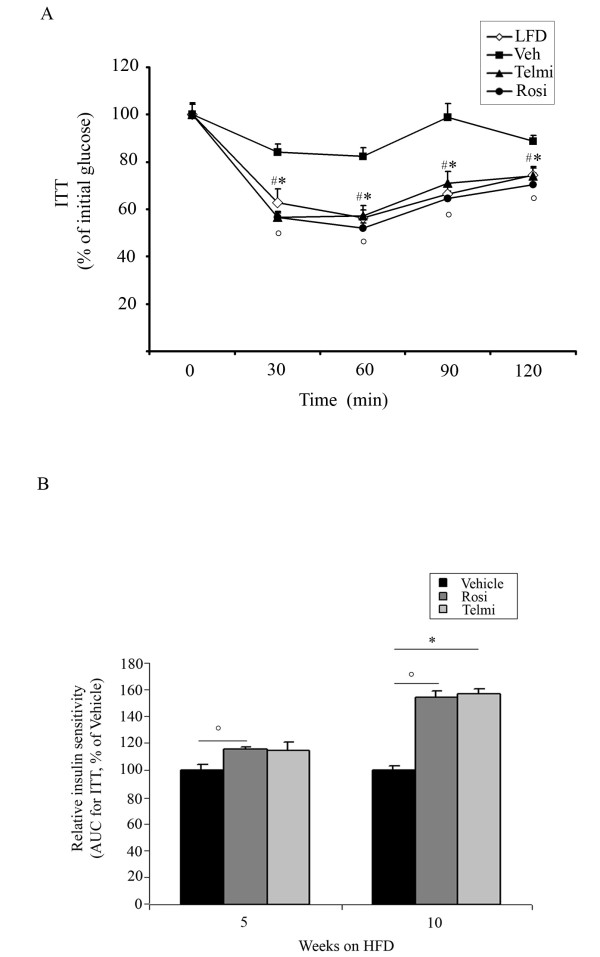
**PPARgamma activation improves systemic and glucose tolerance DIO mice model**. A) Insulin tolerance test (ITT) of LFD-fed, HFD-fed and vehicle-, rosiglitazone-, or telmisartan-treated mice after 10-weeks of intervention, as indicated and described in Methods, n = 10; * p < 0.05 rosiglitazone-treated mice vs. vehicle-treated animals,°p < 0.05 telmisartan-treated mice vs. vehicle-treated animals, # p < 0.05 LFD-fed vs. HFD-fed, vehicle-treated mice. B) Insulin tolerance test (ITT) of HFD-fed, vehicle-, rosiglitazone-, or telmisartan-treated mice after 5- and 10-weeks of intervention, as indicated. From each group area under the curve (AUC) was calculated, as described in Methods, n = 10;°p < 0.05 rosiglitazone-treated mice vs. vehicle-treated animals; * p < 0.05 telmisartan-treated mice vs. vehicle-treated animals.

Increased body weight gain observed in vehicle-treated mice after 5- and 10-weeks of HFD feeding was accompanied with the development of IR and GI, shown in the ipGTT and ITT tests (Figure 1 and 2). Pharmacological intervention with both rosiglitazone and telmisartan led to a significant improvement of GI after 5- and 10-weeks of treatment in our model (Figure 1A and 1B). In accordance, relative insulin sensitivity was significantly increased in mice treated with PPARgamma ligands (Figure 2A and 1B).

These experiments suggest that activation of PPARgamma by the full agonist rosiglitazone as well as the partial agonist telmisartan results in an improvement of HFD-induced metabolic phenotypes.

### 2. Ligand-mediated PPARgamma activation inhibits T-lymphocytes infiltration into in adipose tissue during HFD-induced insulin resistance

**Figure 3:**
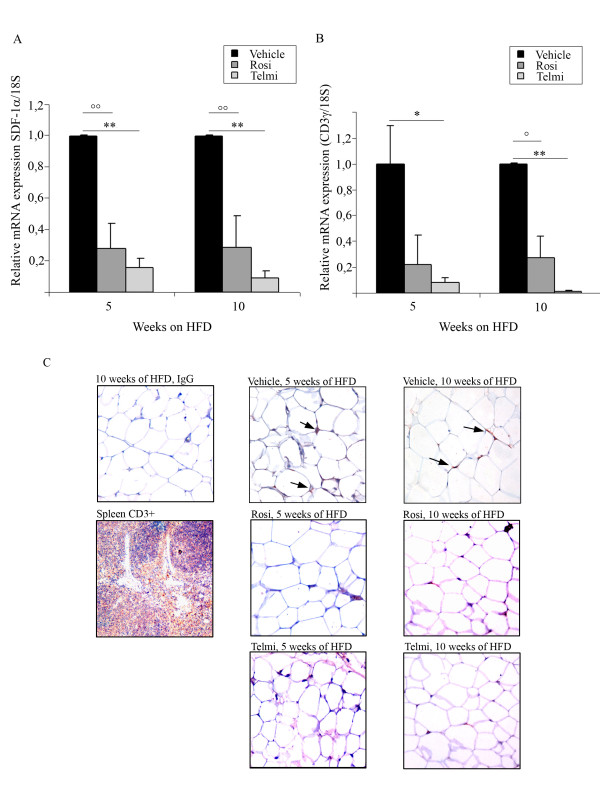
**Ligand-mediated PPARgamma activation inhibits T-lymphocytes infiltration into in adipose tissue during HFD-induced IR**. A) Analysis of SDF-1alpha mRNA expression levels and B) analysis of CD3gamma in abdominal fat from HFD-fed and vehicle-, rosiglitazone-, or telmisartan-treated mice after 5- and 10-weeks of intervention;°°p < 0.01 rosiglitazone-treated mice vs. vehicle-treated animals;°p < 0.05 rosiglitazone-treated mice vs. vehicle-treated animals, ** p < 0.01 telmisartan-treated mice vs. vehicle-treated animals; * p < 0.05 telmisartan-treated mice vs. vehicle-treated animals. C) Immunohistological analysis of the gonadal fat tissue sections isolated from HFD-fed and vehicle-, rosiglitazone-, or telmisartan-treated mice after 5- and 10-weeks of intervention; stained with CD3-specific antibody, as described in Methods. Positive cells are indicated by an arrow. For control staining spleen sections were used. For the negative control the same sections of gonadal adipose tissue isolated from HFD-fed mice were stained with unspecific IgG-antibodies, as indicated.

We and others have recently demonstrated that early IR correlates with the infiltration of T-lymphocytes into adipose tissue[6,15]. To follow this idea we examined the expression of T-lymphocyte marker CD3 in abdominal fat tissue of HFD-fed mice treated with PPARgamma agonists. Expression of T-lymphocyte specific chemoattractants such as SDF-1alpha and T-lymphocyte marker CD3gamma were strongly reduced after 5-and 10-weeks of pharmacological interventions (Figure 3A and 3B). The mRNA expression data were verified by immunohistological analysis of adipose tissue sections from treated- and untreated-obese mice, as shown in Figure 3C. T-lymphocytes could be detected after 5- weeks HFD in vehicle-treated animals. Both PPARgamma ligands completely diminished T-cell staining after 5- and 10-weeks of HFD, indicating that PPARgamma activation prevents T-cell recruitment into adipose tissue during HFD-feeding.

These results indicate a novel putative mechanism of anti-inflammatory action of PPARgamma during early insulin resistance.

### 3. Accumulation of macrophages in adipose tissue is inhibited by PPARgamma activation

**Figure 4:**
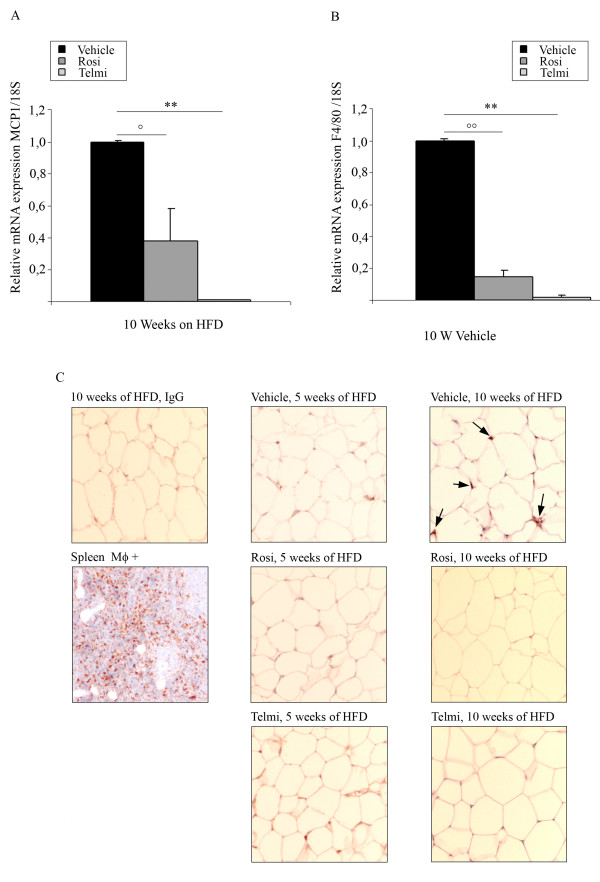
**Accumulation of macrophages in adipose tissue is inhibited by PPARgamma activation**. B) Analysis of MCP-1 mRNA expression levels and B) analysis of F4/80 in abdominal fat from HFD-fed and vehicle-, rosiglitazone-, or telmisartan-treated mice after 10-weeks of intervention;°°p < 0.01 rosiglitazone-treated mice vs. vehicle-treated animals;°p < 0.05 rosiglitazone-treated mice vs. vehicle-treated animals, ** p < 0.01 telmisartan-treated mice vs. vehicle-treated animals. C) Immunohistological analysis of the gonadal fat tissue sections isolated from HFD-fed mice treated with vehicle, rosiglitazone, or telmisartan after 10-weeks of intervention; stained with monoclonal antibody detecting mouse macrophage marker (RM0029-11H3-specific antibody), as described in Methods. Positive cells are indicated by an arrow. As positive control spleen sections were used. For the negative control the same sections of gonadal adipose tissue isolated from HFD-fed mice were with unspecific IgG-antibodies, as indicated.

Accumulation of macrophages in adipose tissue is a central pathogenetic process during the development of diet-induced IR. In consonance, after 10-weeks of HFD, we were able to detect an increased expression of the macrophage marker F4/80, as well as MCP-1, in adipose tissue from vehicle-treated mice (Figure 4A and 3B). These data was accompanied by a positive immunohistological staining for macrophages (RM0029-11H3) in adipose tissue sections of vehicle-treated HFD-fed mice (Figure 4C). The expression of both F4/80 and MCP-1 was strongly reduced in adipose tissue from rosiglitazone- and telmisartan-treated- HFD-fed mice (Figure 4A). We could also confirm our findings by immunohistological analysis with RM0029-11H3-specific staining (Figure 4B). Interestingly, macrophage staining and marker mRNA (F4/80, MCP-1) expression was absent in 5- weeks vehicle-treated HFD animals corroborating that macrophages infiltration is likely a later event in diet-induced IR.

Taken together our data illustrate a new anti-inflammatory action of PPARgamma regarding anti-inflammation, which may have an impact on the development and progression of IR. Our experiments demonstrate that macrophage infiltration could be specifically reduced by the intervention with PPARgamma agonists.

## Discussion

The present study demonstrates that activation of PPARgamma reduces HFD-induced T-lymphocyte infiltration into adipose tissue. Both PPARgamma agonists, rosiglitazone and telmisartan, were able to reduce diet-induced T-lymphocyte accumulation in fat tissue. Subsequently, PPARgamma activation was linked to reduced macrophage infiltration into adipose tissue of HFD-fed mice. Anti-inflammatory actions of PPARgamma in adipose tissue were associated with an improved metabolic phenotype of mice treated with PPARgamma agonists for 5- and 10-weeks parallel to the HFD-intervention. T-lymphocytes were recently shown to initiate the inflammatory response associated with obesity-related IR. Our data demonstrate for the first time a new anti-inflammatory action of PPARgamma agonists targeting T-cells, which may contribute to the beneficial metabolic actions of PPARgamma ligands.

The ARB telmisartan has been shown to improve metabolic outcome of DIO-mice [13]. Although the molecular mechanism of telmisartan´s action is not fully understood, its beneficial metabolic properties are linked to the ability to activate PPARgamma as a partial agonist, similarly to potent anti-diabetic drugs such as TZDs [13,16]. Telmisartan has also been demonstrated to modulate hepatic mitochondrial fatty acid oxidation due to the activation of hepatic PPARalpha[17]. Along that line, a recently published study from Rong. X and colleagues on AT1R-deficient mice indicates for the first time that beneficial effects of telmisartan on diet-induced obesity, insulin resistance and hepatic triglyceride accumulation in mice appear to be AT1-R independent [18].

Recent work published by Hernandez-Trujillo and colleagues [19] demonstrated that the administration of rosiglitazone or losartan to mice fed a high-fat, high-cholesterol diet increases PPARgamma activity in both cases. Interestingly, treatment with rosiglitazone showed anti-atherogenic properties, such as a significant reduction of eNOS and CD36 expression, when compared to littermates treated with losartan. Furthermore, rosiglitazone has been shown to improve endothelial dysfunction in femoral arteries of Zucker diabetic fatty rats, mainly due to the regulation of eNOS expression and enhancement of endothelium-dependent vasorelaxation. Rosiglitazone treatment did not affect the mechanical properties of the artery as well as an expression of alpha-adrenoceptor (alpha-AR), MMP9 and elastase in this model [20].

Importantly, treatment with both rosiglitazone and telmisartan led to the reduction of BW after 10 weeks of HFD, when compared to vehicle-treated littermates. Pair-feeding protocols reduced but not fully eliminated the differences in BW observed in HFD-fed mice treated with rosiglitazone and telmisartan. The BW difference can potentially have an impact on the anti-inflammatory and anti-diabetic properties of those two PPARgamma agonists.

In previous studies we characterized the role of T-lymphocytes in adipose tissue inflammation[6]. Lymphocyte infiltration occurs during the early phases of IR development in HFD-induced obesity and precedes adipose tissue macrophage accumulation. Furthermore, it was demonstrated by us and the others, that activation of PPARgamma by TZDs or telmisartan reduces inflammatory T-cell activity, C-peptide-induced T-cell chemotaxis, and SDF-1- directed T-cell migration in vitro[21-24], indicating an important function of PPARgamma in T-cells. Along this line, our present study shows that ligand-mediated PPARgamma activation prevents T-cell infiltration into adipose tissue which was associated with an improvement of diet-induced IR.

In their recent study Winer and colleagues identified, similar to our data, a residential population of T4-lymphocytes that controlled insulin resistance in visceral adipose tissue of HFD-fed mice. In elegant transplantation experiments with CD4^+ ^T-lymphocyte transfer into lymphocyte-free Rag1-null- and HFD-fed mice the authors showed reversed weight gain and improved IR of these mice in comparison to control littermates predominantly mediated through anti-inflammatory T_H_2 cells[15]. In addition, a distinct set of the CD4^+ ^and Foxp3^+^- T-cells (T-regs) were recently identified in adipose tissue from lean rodents. These cells have been also suggested to play a regulatory role of the metabolic phenotype modulating an anti-inflammatory response in adipose tissue[25]. Accordingly, the population of T-reg cells was dramatically reduced in adipose tissue of mice with obesity-related IR confirming a beneficial function of this T-cell population. It appears that a pro- and anti-inflammatory subset of CD4^+ ^T-lymphocytes does exist in adipose tissue, and the balance betweens these populations likely determines the phenotype. In our study we did not investigate CD4^+ ^T-lymphocyte subsets; however, as previously described in other studies using the HFD model, pro-inflammatory T_H_1 cells are the predominant subset during weight gain. Therefore it is likely that PPARgamma ligands mainly target T_H_1 cells in our model to mediate their anti-inflammatory action.

In addition to the reduction of T-lymphocyte accumulation by PPARgamma ligands we demonstrate that the amount of macrophages is reduced in groups treated with rosiglitazone or telmisartan. Accordingly to the characterization of CD4^+ ^T-lymphocyte subsets in adipose tissue, distinct subpopulations (pro-inflammatory M1 and anti-inflammatory M2) of macrophages have been identified in adipose tissue[3,10]. PPARgamma has been recently demonstrated to promote infiltration of M2 (anti-inflammatory) macrophages into adipose tissue in DIO model in mice[10]. In this work mice were fed with HFD over 20-weeks, followed by 1-week treatment with rosiglitazone (0.01% w/w) the authors reported a striking up-regulation of the MCP-1 expression in the adipose tissue of rosiglitazone-treated mice, which is in contrast to our data, as well as to the results in human and murine adipose tissue published by others[5,26]. This discrepancy between our data and results published by Stienstra and colleagues could be explained, at least in part, by the different DIO model used in the study. Stienstra and colleagues investigated chronic fat tissue inflammation followed by a short pharmacological intervention with rosiglitazone. We were interested in the early stages of IR development, and mice used in our work were set on HFD parallel to the rosiglitazone/telmisartan intervention for over 10-weeks. In our mice model we could observe a clear reduction of adipose tissue MCP-1 by PPARgamma agonists, which is in agreement with previously published data[5]. Stienstra and collaborators observed also enhanced infiltration of M2 macrophages after rosiglitazone intervention, when compared to vehicle-treated animals. This is in agreement with data published by Bassaganya-Riera and others[9], showing that deficiency of PPARgamma in immune cells results in significant upregulation of the M1 markers and repression of M2 markers characteristic for macrophages in white adipose tissue of mice with HFD-induced obesity. Lack of PPARgamma led to the dominance of M1 differentiation in this model. Following this line, reduction of MCP-1 and macrophage markers by PPARgamma ligands in our study likely reflect the diminution of pro-inflammatory M1-macrophages during the early development of obesity/IR. Enhancement of M2-macrophage responses by PPARgamma activation might be a feature of late phase during HFD feeding.

Although our study clearly indicate that macrophage infiltration and the progression of fat tissue inflammation and IR could be specifically reduced by the intervention with PPARgamma agonists, one has to take into account that conclusion derived form animal studies in general cannot be automatically extrapolated to human or to clinical studies, as treatment with rosiglitazone was recently linked with increased cardiovascular risk [27].

## Conclusion

In summary, we have shown for the first time the inhibitory properties of PPARgamma activation on T-lymphocyte-derived adipose tissue inflammation. Our data indicate a novel mechanism of PPARgamma function in the development of IR, acting as a potent anti-inflammatory factor on T-lymphocyte activation and infiltration into fat tissue, and by this mean contributing to the attenuation of the systemic development of IR.

## Competing interests

NM, TU, UK have received research grants and/ or speaker fees from GSK, BI, Bayer.

## Authors' contributions

AFL contributed to research data, contributed to discussion, wrote and edited MS, MH contributed to research data and edited MS, MC contributed to research data, CS contributed to research data, KH contributed to research data, NM contributed to discussion, reviewed and edited manuscript, TU contributed to discussion, reviewed and edited manuscript and UK designed the study, wrote the MS, contributed to discussion, reviewed and edited manuscript. All authors read and approved the final manuscript.
